# Childhood cancer incidence and survival in Japan and England: A population‐based study (1993‐2010)

**DOI:** 10.1111/cas.13457

**Published:** 2017-12-26

**Authors:** Kayo Nakata, Yuri Ito, Winnie Magadi, Audrey Bonaventure, Charles A. Stiller, Kota Katanoda, Tomohiro Matsuda, Isao Miyashiro, Kathy Pritchard‐Jones, Bernard Rachet

**Affiliations:** ^1^ Cancer Control Center Osaka International Cancer Institute Osaka Japan; ^2^ Developmental Biology and Cancer Programme UCL Great Ormond Street Institute of Child Health University College London London UK; ^3^ Cancer Survival Group London School of Hygiene & Tropical Medicine London UK; ^4^ National Cancer Registration and Analysis Service Public Health England Oxford UK; ^5^ Center for Cancer Control and Information Services National Cancer Center Tokyo Japan

**Keywords:** cancer registry data, childhood cancer, childhood cancer incidence and survival, epidemiology, population‐based study

## Abstract

The present study aimed to compare cancer incidence and trends in survival for children diagnosed in Japan and England, using population‐based cancer registry data. The analysis was based on 5192 children with cancer (age 0‐14 years) from 6 prefectural cancer registries in Japan and 21 295 children diagnosed in England during 1993‐2010. Differences in incidence rates between the 2 countries were measured with Poisson regression models. Overall survival was estimated using the Kaplan–Meier method. Incidence rates for Hodgkin lymphoma, renal tumors and Ewing sarcomas in England were more than twice as high as those in Japan. Incidence of germ cell tumors, hepatic tumors, neuroblastoma and acute myeloid leukemia (AML) was higher in Japan than in England. Incidence of all cancers combined decreased in Japan throughout the period 1993 to 2010, which was mainly explained by a decrease in registration of neuroblastoma in infants. For many cancers, 5‐year survival improved in both countries. The improvement in survival in chronic myeloid leukemia (CML) was particularly dramatic in both countries. However, 5‐year survival remained less than 80% in 2005‐2008 in both countries for AML, brain tumors, soft tissue sarcomas, malignant bone tumors and neuroblastoma (age 1‐14 years). There were significant differences in incidence of several cancers between countries, suggesting variation in genetic susceptibility and possibly environmental factors. The decrease in incidence for all cancers combined in Japan was related to the cessation of the national screening program for neuroblastoma. The large improvement in survival in CML coincided with the introduction of effective therapy (imatinib).

## 1 INTRODUCTION

Every year approximately 215 000 children (aged 0‐14 years) are diagnosed with cancer globally, while 80 000 children die from the disease.^1^ Epidemiological analyses of differences in incidence and survival between countries and over time are important to understand etiological factors and to monitor changes in disease burden and progress in the treatment of childhood cancers.

The incidence of childhood cancer overall and by diagnostic subgroup has been reported in the International Incidence of Childhood Cancer (IICC) for many countries, including Japan and England.^2^, ^3^ In Europe, survival analysis has been performed to evaluate the quality of care for children with cancer in each country or region in several studies, including the Automated Childhood Cancer Information System (ACCIS)^4^ and EUROCARE‐5.^5^ In 2012, the global surveillance of cancer survival program (the CONCORD‐2 study),^6^, ^7^ which includes childhood leukemia, was initiated using population‐based cancer registry data from 67 countries. In Japan, population‐based studies for childhood cancer comparisons to other countries are scarce, although some recent studies show childhood cancer incidence^8^, ^9^ or survival,^10^ and several cancer registries contributed to the IICC and CONCORD‐2 studies.^2^, ^7^ In England, population‐based incidence and survival for childhood cancer have been reported since 1980s.^11^, ^12^, ^13^ In the current study, we compared incidence and time trends in survival for childhood cancer between Japan and England during the period 1993‐2010, to gain insight into the progress against childhood cancer in both countries.

## 2 METHODS

### 2.1 Data

This study was based on data from population‐based cancer registries in Japan and England. It included all children (0‐14 years) diagnosed with cancer between 1993 and 2010 residing in 6 Japanese prefectures (Miyagi, Yamagata, Niigata, Fukui, Osaka and Nagasaki)^10^ or in England. Japanese data were obtained from the Monitoring of Cancer Incidence in Japan (MCIJ) project and the Japanese Cancer Survival Information for Society (J‐CANSIS) project,^10^ while data for England were obtained from the Office for National Statistics. A standard set of variables included basic demographic data (age, sex and country), information on the tumor (date of diagnosis, site and morphology) and on follow‐up (date of last contact and vital status). Follow‐up information was available at least 5 years after diagnosis in Japan although the patient follow‐up system differs for each cancer registry.^10^ Within the Japanese data, vital status information was available for patients diagnosed during 1993‐2008. In the English data, the vital status was last updated on 31 December 2015.

We included only records of malignant cancers (behavior code/3) defined in the International Classification of Disease for Oncology, 3rd edition (ICD‐O‐3).^14^ Non‐malignant or borderline central nervous system tumors such as craniopharyngioma, meningioma, ganglioglioma, benign teratoma and pilocytic astrocytoma were all excluded. Skin carcinomas were also excluded. Cancers were grouped into 12 main diagnostic categories according to the International Classification of Childhood Cancer, 3rd edition (ICCC‐3).^15^ We modified some subgroups of ICCC‐3, based on the topography and morphology codes from ICD‐O‐3 (Table S1).

These data partially overlapped with the data used in the IICC or CONCORD‐2, although for participating registries or study periods, inclusion criteria were not completely matched. There were few discrepancies in the incidence of each cancer between both datasets, with the exception of central nervous system (CNS) tumors and all cancers combined.

### 2.2 Statistical analysis

Incidence rates were calculated as the average annual number of children newly diagnosed with cancer per million children. Age‐standardized incidence rates (ASR) were calculated by the direct method, using the weights of the world standard population for the age groups under 15 years (0, 1‐4, 5‐9 and 10‐14 years).^16^, ^17^ Changes in incidence rates over time were calculated using a Poisson regression model, divided into 3 time periods (1993‐1998, 1999‐2004 and 2005‐2010) and adjusted for age‐group, and expressed as average annual percent change (AAPC). Differences in incidence rates between the 2 countries were measured with Poisson regression models and expressed as the incidence rate ratio (IRR), using English data as the reference. These ratios were adjusted for time period and age group. Observed population‐based survival was estimated by cancer type in each time period (1993‐1996, 1997‐2000, 2001‐2004 and 2005‐2008), using the Kaplan–Meier method. We used the classic cohort approach to calculate 1‐year and 5‐year survival for children diagnosed during 1993‐2008, and 10‐year survival for children diagnosed during 1993‐2000 in Japan and during 1993‐2004 in England. We used the period approach to predict 10‐year survival for children diagnosed during 2001‐2008 in Japan and during 2005‐2008 in England, as this approach allows for the prediction of survival where 10‐year follow‐up is not yet available.^18^ The analysis was carried out using Stata 14. This study was approved by the London‐South East Research Ethics Committee (07/MRE01/52) and the Research Ethics Committee of the Osaka International Cancer Institute (No. 1707105096).

## 3 RESULTS

### 3.1 Data quality

Analyses were based on 5192 cases in Japan and 21 295 cases in England between 1993 and 2010. Table 1 shows the quality criteria for validity and completeness of the data over time in each country. The proportion of records from death certificate only (DCO) in Japan reduced from 3.1% in 1993‐1998 to 0.9% in 2005‐2010, whereas that in England has been stable at under 1% since 1993. The proportion of unspecified histology (not otherwise specified [NOS], ICD‐O‐3 morphology code 8000 to 8004) also decreased from 4.3 to 2.1% in Japan, whereas that in England was around 2% from 1993 to 2010.^19^ The proportion of multiple primary cancers was under 1% in both countries, except in the Japanese data for 2005‐2010. We included NOS for both incidence and survival analysis while DCO and multiple primary cancers were excluded in survival analysis.

**Table 1:** Indicators of data quality of population‐based cancer registries between Japan and England

	Records	Unspecified histology^a^		DCO^b^		Multiple primary cancers^b^	
	N	N	%	N	%	N	%
Japan (6 cancer registries)							
1993‐1998	1947	84	4.3	60	3.1	15	0.8
1999‐2004	1704	74	4.3	21	1.2	15	0.9
2005‐2010	1541	32	2.1	14	0.9	33	2.1
England							
1993‐1998	7019	152	2.2	38	0.5	35	0.5
1999‐2004	7087	101	1.4	14	0.2	49	0.7
2005‐2010	7189	169	2.4	14	0.2	50	0.7

DCO, records registered from death certificate only.

a ICD‐O‐3 morphology code 8000 to 8004.

b We included for incidence analysis, but excluded for survival analysis.

### 3.2 Trends in incidence of childhood cancer in Japan and England

Table 2 shows trends in incidence for each cancer type in both countries. Overall, the age‐standardized incidence rate (ASR) of all childhood cancers combined seemed to decrease in Japan (ASR 1993‐1998: 127 per million vs 2005‐2010: 116 per million; see Table 2 and Figure S1A). However, incidence for all cancers except neuroblastoma was stable (AAPC = 0.2%, [95% CI −0.4‐0.8], Table 2). A steep decline was observed in neuroblastoma (NBL) in infants (age <1 year) in Japan (average age‐specific incidence rate changed from 191 to 27 per million; Table 2, Figure S1B). In England, the incidence of all childhood cancers increased from 1993‐1998 to 1999‐2004, and plateaued (ASR 1993‐1998:129, 1999‐2004: 133, 2005‐2010:134). Incidence increased significantly during 1993‐2010 in England for malignant bone tumors (AAPC = 1.3%, [95% CI 0.1‐2.5]) and germ cell tumors (GCT; AAPC = 1.6%, [0.1‐3.1]), but not for the other cancer types.

**Table 2:** Trends in incidence of childhood cancer (age 0‐14 y) by period of diagnosis and cancer type in Japan and England

	1993‐1998			1999‐2004			2005‐2010			Time trend	
	N	ASR	[95% CI]	N	ASR	[95% CI]	N	ASR	[95% CI]	AAPC	[95% CI]
Japan											
I. Leukemias	670	43.2	[39.9‐46.6]	558	39.6	[36.3‐43]	566	43.0	[39.4‐46.6]	0.1	[−1.1‐0.8]
II. Lymphomas	197	12.3	[10.5‐14]	158	10.4	[8.8‐12]	154	10.7	[9‐12.4]	−0.7	[−2.5‐1.1]
III. CNS tumors	290	18.3	[16.1‐20.4]	257	17.5	[15.4‐19.7]	249	18.3	[16‐20.6]	0.1	[−1.3‐1.5]
IV. Neuroblastoma	282	21.2	[18.7‐23.7]	219	17.4	[15.1‐19.7]	103	9.0	[7.2‐10.7]	−6.4	[−8.1‐4.7]
NBL infants (age < 1 y)	192	191.3^a^		121	128.4^a^		23	27.3^a^		n.a.	
NBL children aged 1‐14 y	90	6.9^b^	[5.5‐8.4]	98	8.1^b^	[6.5‐9.7]	80	7.4^b^	[5.8‐9]	1.3	[−2.2‐2.7]
V. Retinoblastoma	56	4.3	[3.1‐5.4]	57	4.6	[3.4‐5.7]	57	5.1	[3.8‐6.5]	1.5	[−1.6‐4.5]
VI. Renal tumors	52	3.8	[2.7‐4.8]	47	3.5	[2.5‐4.5]	36	3.1	[2.1‐4.1]	−1.7	[−5.2‐1.8]
VII. Hepatic tumors	36	2.6	[1.7‐3.4]	40	3.1	[2.1‐4.1]	37	3.2	[2.2‐4.2]	1.6	[−2.2‐5.3]
VIII. Malignant Bone tumors	83	4.4	[3.4‐5.3]	83	4.9	[3.9‐6]	67	4.3	[3.3‐5.3]	−0.1	[−2.7‐2.5]
IX. Soft tissue sarcomas	96	5.9	[4.7‐7.1]	104	7.2	[5.8‐8.6]	95	6.8	[5.4‐8.2]	1.3	[−1‐3.7]
X. Germ cell tumors	128	7.6	[6.2‐8.9]	113	7.4	[6‐8.7]	118	8.5	[6.9‐10]	0.8	[−1.3‐2.9]
XI. Other carcinomas	42	2.2	[1.5‐2.9]	47	2.8	[2‐3.6]	43	2.8	[1.9‐3.6]	1.8	[−1.6‐5.3]
XII. Unspecified cancers	15	1.0	[0.5‐1.5]	21	1.5	[0.8‐2.1]	16	1.2	[0.6‐1.8]	1.9	[−3.7‐7.4]
Total	1947	126.6	[120.9‐132.3]	1704	119.9	[114.1‐125.6]	1541	115.9	[110‐121.8]	−0.6	[−1.1‐0]
Total (excluding NBL)	1665	105.4	[100.2‐110.5]	1485	102.5	[97.2‐107.8]	1438	107.0	[101.3‐112.6]	0.2	[−0.4‐0.8]
England											
I. Leukemias	2462	45.8	[43.9‐47.6]	2493	48.0	[46.1‐49.9]	2436	46.6	[44.7‐48.4]	0.1	[−0.3‐0.5]
II. Lymphomas	802	13.7	[12.8‐14.7]	815	13.7	[12.8‐14.7]	864	14.7	[13.7‐15.7]	0.7	[−0.1‐1.5]
III. CNS tumors	1252	22.6	[21.4‐23.9]	1241	22.9	[21.6‐24.2]	1226	22.8	[21.5‐24.1]	0.0	[−0.6‐0.7]
IV. Neuroblastoma	476	9.4	[8.6‐10.3]	468	10.0	[9.1‐10.9]	461	9.3	[8.4‐10.1]	−0.2	[−1.2‐0.9]
NBL infants (age <1 y)	128	34.7^a^		157	45.4^a^		149	38.5^a^		n.a.	
NBL children aged 1‐14 y	348	7.30^b^	[6.5‐8.1]	311	7.0^b^	[6.2‐7.8]	312	6.8^b^	[6.1‐7.6]	−0.6	[−1.9‐0.7]
V. Retinoblastoma	225	4.6	[4‐5.2]	203	4.4	[3.8‐5.1]	219	4.5	[3.9‐5.1]	−0.2	[−1.8‐1.3]
VI. Renal tumors	434	8.5	[7.7‐9.3]	449	9.3	[8.5‐10.2]	449	9.0	[8.2‐9.9]	0.5	[−0.6‐1.6]
VII. Hepatic tumors	74	1.5	[1.1‐1.8]	93	1.9	[1.5‐2.3]	89	1.8	[1.4‐2.1]	1.5	[−1‐3.9]
VIII. Malignant bone tumors	318	5.2	[4.7‐5.8]	355	5.7	[5.1‐6.3]	371	6.1	[5.5‐6.7]	1.3	[0.1‐2.5]
IX. Soft tissue sarcomas	486	8.9	[8.1‐9.6]	470	8.6	[7.8‐9.4]	493	9.0	[8.2‐9.8]	0.2	[−0.8‐1.3]
X. Germ cell tumors	221	4.0	[3.5‐4.5]	233	4.2	[3.6‐4.7]	270	4.8	[4.2‐5.4]	1.6	[0.1‐3.1]
XI. Other carcinomas	201	3.4	[2.9‐3.8]	215	3.5	[3‐4]	230	3.8	[3.3‐4.3]	1.0	[−0.5‐2.6]
XII. Unspecified cancers	68	1.2	[1‐1.5]	52	1.0	[0.7‐1.3]	81	1.5	[1.2‐1.8]	1.7	[−1.1‐4.5]
Total	7019	128.8	[125.7‐131.8]	7087	133.3	[130.2‐136.5]	7189	133.8	[130.7‐136.9]	0.3	[0.1‐0.6]
Total (excluding NBL)	6543	119.4	[116.4‐122.3]	6619	123.3	[120.3‐126.3]	6728	124.5	[121.5‐127.5]	0.4	[0.1‐0.7]

AAPC, average annual percentage of change; ASR, age‐standardized incidence rate (person per million‐years); CNS, central nervous system; NBL, neuroblastoma; n.a., AAPCs of “NBL infants (age < 1 y)” were not calculated because models were not fitted.

a Age‐specific incidence rate.

b Using records, population and world standard population in age 1‐4, 5‐9, 10‐14 y.

### 3.3 Comparison of incidence of each cancer type between countries

**Figure 1:**
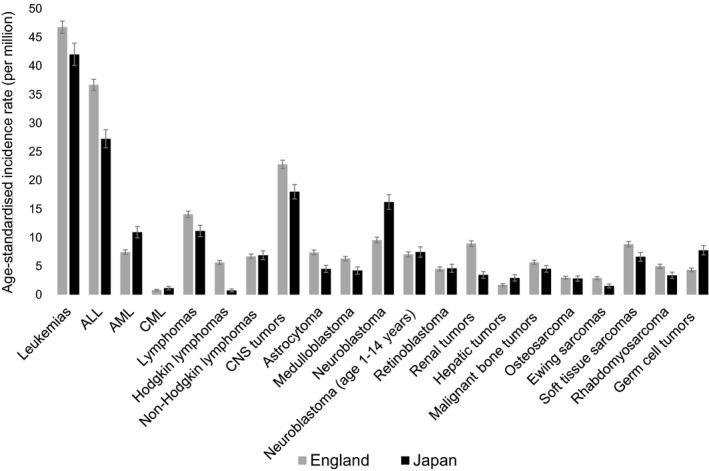
Age‐standardized incidence rate of childhood cancer (age 0‐14 y) by cancer type in Japan and England, 1993‐2010. The error bars indicate the 95% confidence intervals. ALL, acute lymphoblastic leukemias; AML, acute myeloid leukemias; CML, chronic myeloid leukemia; CNS, central nervous system; NBL, neuroblastoma

Table 3 and Figure 1 show ASR in the total period of 1993‐2010 and the incidence rate ratios (IRR) for each cancer type between Japan and England (England reference), adjusted for time period and age group. Incidence rates for leukemias (IRR = 0.9, [95% CI 0.9‐1.0], *P* < .01), acute lymphoblastic leukemias (ALL) (IRR = 0.8, [0.7‐0.8], *P* < .01), lymphomas (IRR = 0.7, [0.7‐0.8], *P* < .01), Hodgkin lymphomas (HL; IRR = 0.1, [0.1‐0.2], *P* < .01), malignant CNS tumors (IRR = 0.8, [0.7‐0.8], *P* < .01), astrocytoma (IRR = 0.6, [0.6‐0.7], *P* < .01), medulloblastoma (IRR = 0.7, [0.6‐0.8], *P* < .01), renal tumors (IRR = 0.4, [0.3‐0.5], *P* < .01), malignant bone tumors (IRR = 0.8, [0.7‐0.9], *P* < .01), Ewing sarcoma family of tumors (Ewing sarcomas, in both bone and soft tissue; IRR = 0.5, [0.4‐0.7], *P* < .01), soft tissue sarcomas (IRR = 0.8, [0.7‐0.9], *P* < .01), rhabdomyosarcomas (IRR = 0.7, [0.6‐0.8], *P* < .01) and other carcinomas (IRR = 0.7, [0.6‐0.9], *P* < .01) were significantly higher in England than Japan. Moreover, incidence rates for HL, renal tumors and Ewing sarcomas in England were over twice as high as those in Japan. Incidence rates for acute myeloid leukemias (AML; IRR = 1.5, [1.3‐1.6], *P* < .01), chronic myeloid leukemias (CML, IRR = 1.4, [1.0‐1.9], *P* = .044), NBL (IRR = 1.7, [1.5‐1.8], *P* < .01), hepatic tumors (IRR = 1.7, [1.4‐2.1], *P* < .01) and GCT (IRR = 1.8, [1.6‐2.1], *P* < .01) were significantly higher in Japan than England. Incidence rates for non‐Hodgkin lymphomas (NHL), NBL (children aged 1‐14 years), retinoblastoma, osteosarcomas and soft tissue sarcomas (excluding RMS and Ewing sarcomas) were similar in Japan and England, and the differences were non‐significant. Incidence rates of unspecified subtypes in each cancer group were higher in Japan than in England for leukemias (IRR = 2.1, [1.6‐2.7], *P* < .01), lymphomas (IRR = 1.7, [1.3‐2.2], *P* < .01), CNS tumors (IRR = 2.8, [2.2‐3.5], *P* < .01) and GCT (IRR = 2.2 [1.1‐4.5], *P* = .02).

**Table 3:** Age‐standardized incidence rate (ASR) and incidence rate ratio (IRR, England reference) of childhood cancer (age 0‐14 y) in Japan and England, 1993‐2010

	Japan			England			IRR	[95%CI]	*P*‐value
	N	ASR	[95%CI]	N	ASR	[95%CI]			
I. Leukemias	1794	42.0	[40‐44]	7391	46.8	[45.7‐47.8]	0.9	[0.9‐1]	<.01
ALL	1156	27.3	[25.7‐28.8]	5766	36.7	[35.7‐37.6]	0.8	[0.7‐0.8]	<.01
AML	474	10.9	[9.9‐11.9]	1203	7.4	[7‐7.9]	1.5	[1.3‐1.6]	<.01
CML	53	1.1	[0.8‐1.5]	139	0.8	[0.7‐0.9]	1.4	[1‐1.9]	.044
Unspecified leukemias	81	2.0	[1.5‐2.4]	147	0.9	[0.8‐1.1]	2.1	[1.6‐2.7]	<.01
II. Lymphomas	509	11.1	[10.2‐12.1]	2481	14.1	[13.5‐14.6]	0.8	[0.7‐0.8]	<.01
Hodgkin lymphomas	38	0.8	[0.5‐1]	1039	5.6	[5.3‐6]	0.1	[0.1‐0.2]	<.01
Non‐Hodgkin lymphomas^a^	321	6.9	[6.1‐7.7]	1157	6.7	[6.3‐7.1]	1.0	[0.9‐1.2]	.7
Unspecified lymphomas	92	2.0	[1.6‐2.4]	188	1.1	[0.9‐1.3]	1.7	[1.3‐2.2]	<.01
III. CNS tumors	796	18.0	[16.7‐19.3]	3719	22.8	[22‐23.5]	0.8	[0.7‐0.9]	<.01
Astrocytoma	210	4.5	[3.9‐5.1]	1222	7.4	[7‐7.8]	0.6	[0.6‐0.7]	<.01
Medulloblastoma	184	4.2	[3.6‐4.9]	1031	6.3	[5.9‐6.7]	0.7	[0.6‐0.8]	<.01
Unspecified CNS tumors	129	2.9	[2.4‐3.4]	173	1.1	[0.9‐1.2]	2.8	[2.2‐3.5]	<.01
IV. Neuroblastoma	604	16.2	[14.9‐17.5]	1405	9.6	[9.1‐10.1]	1.7	[1.5‐1.8]	<.01
NBL children aged 1‐14 y	268	7.5	[6.6‐8.4]	971	7.1	[6.6‐7.5]	1.1	[0.9‐1.2]	.46
V. Retinoblastoma	170	4.6	[3.9‐5.3]	647	4.5	[4.2‐4.9]	1.0	[0.9‐1.2]	.78
VI. Renal tumors	135	3.5	[2.9‐4.1]	1332	8.9	[8.5‐9.4]	0.4	[0.3‐0.5]	<.01
VII. Hepatic tumors	113	2.9	[2.4‐3.5]	256	1.7	[1.5‐1.9]	1.7	[1.4‐2.1]	<.01
VIII. Malignant bone tumors	233	4.5	[3.9‐5.1]	1044	5.7	[5.3‐6]	0.8	[0.7‐0.9]	<.01
Osteosarcomas	148	2.8	[2.4‐3.3]	558	3.0	[2.7‐3.2]	1.0	[0.8‐1.1]	.57
Ewing sarcomas (bone and soft tissue)^b^	76	1.5	[1.2‐1.9]	518	2.9	[2.6‐3.1]	0.5	[0.4‐0.7]	<.01
Unspecified malignant bone tumors	9	0.2	[0.1‐0.3]	49	0.3	[0.2‐0.4]	0.7	[0.3‐1.4]	.26
IX. Soft tissue sarcomas	295	6.6	[5.9‐7.4]	1449	8.8	[8.4‐9.3]	0.8	[0.7‐0.9]	<.01
Rhabdomyosarcomas (RMS)	148	3.4	[2.8‐3.9]	788	5.0	[4.6‐5.3]	0.7	[0.6‐0.8]	<.01
Soft tissue sarcomas (excluding RMS and Ewing sarcomas)	124	2.8	[2.3‐3.3]	536	3.1	[2.9‐3.4]	0.9	[0.7‐1]	.122
Unspecified soft tissue sarcomas	22	0.5	[0.3‐0.7]	127	0.7	[0.6‐0.9]	0.6	[0.4‐1.0]	.05
X. Germ cell tumors	359	7.8	[7‐8.6]	724	4.3	[4‐4.7]	1.8	[1.6‐2.1]	<.01
Unspecified malignant gonadal tumors	13	0.3	[0.1‐0.4]	21	0.1	[0.1‐0.2]	2.2	[1.1‐4.5]	.02
XI. Other carcinomas	132	2.6	[2.1‐3]	646	3.6	[3.3‐3.8]	0.7	[0.6‐0.9]	<.01
XI. Unspecified cancers	52	1.2	[0.9‐1.5]	201	1.2	[1.1‐1.4]	n.a.		
Total	5192	121.1	[117.8‐124.5]	21295	132.0	[130.2‐133.7]	0.9	[0.9‐0.9]	<.01

ALL, acute lymphoblastic leukemias; AML, acute myeloid leukemias, CML, chronic myeloid leukemia; CNS, central nervous system; NBL, neuroblastoma. IRR were adjusted for time period and age group (using England as the reference). n.a., IRR of “unspecified cancers” (ICCC‐3 group XII) were not calculated because models were not fitted.

a This includes Burkitt lymphoma.

b Ewing sarcoma family of tumors, in both bone and soft tissue.

We analyzed age‐specific incidence rates by sex for some solid tumors (Wilms tumor, hepatoblastoma and GCT of each site) in each country (Table S2 and Figure S2). The peak age for Wilms tumor in Japan was infants aged under 1 year, whereas in England it was children aged 1‐4 years. Hepatoblastoma was the most common type of hepatic tumor in both countries (N = 100 [88%] in Japan vs N = 211 [82%] in England) and the age distribution was similar between countries. Age‐specific incidence rates for intracranial GCT were higher in Japan than in England for all age groups. Incidence of gonadal GCT in male infants in Japan was much higher than in England. However, the numbers were too small to perform any relevant statistical comparison.

### 3.4 Trends in survival for each cancer type in Japan and England

**Figure 2:**
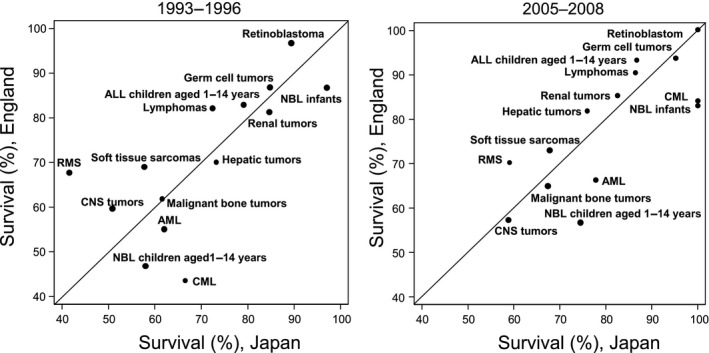
Five‐year survival for each cancer type in Japan and England, 1993‐1996 and 2005‐2008. Five‐year survival for some major diagnostic groups in Japan and England are plotted on the same graphic for each period. ALL, acute lymphoblastic leukemias; AML, acute myeloid leukemias; CML, chronic myeloid leukemia; CNS, central nervous system; NBL, neuroblastoma; RMS, rhabdomyosarcoma

**Figure 3:**
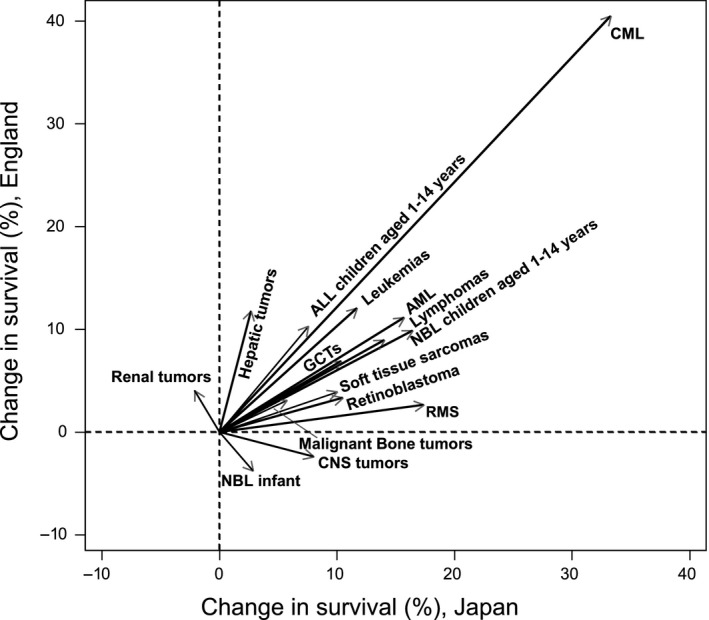
Change in 5‐y survival for each cancer type from 1993‐1996 to 2005‐2008, in Japan and England. ALL, acute lymphoblastic leukemias; AML, acute myeloid leukemias; CML, chronic myeloid leukemia; CNS, central nervous system GCTs, germ cell tumors; NBL, neuroblastoma; RMS, rhabdomyosarcoma

We analyzed trends in 1‐year, 5‐year and 10‐year survival for each cancer type and each period in both countries (Table4). One‐year survival was over 80% in most cancers in both countries in the period 2005‐2008, except for hepatic tumors (76%) in Japan and AML (79%) and CNS tumors (75%) in England. Five‐year survival for leukemias significantly improved from 1993‐1996 to 2005‐2008, reaching over 80% in 2005‐2008 in both countries (Japan: 71% [95% CI 67‐75] to 83% [79‐86], England: 76% [74‐78] to 88% [86‐90]), whereas 5‐year survival for CNS tumors remained at 50% in both countries. Ten‐year survival was over 80% for ALL (children aged 1‐14 years), CML, lymphomas, NBL infants (age <1 year), retinoblastoma, renal tumors and germ cell tumors in both countries. To calculate 10‐year survival in recent periods, we used a different approach (period approach) from the cohort approach, so there was divergence between 5‐year survival and 10‐year survival (higher survival in 10‐year survival than 5‐year survival) in some cancers (lymphomas, NBL, renal tumors, and unspecified cancers in Japan, and AML, CML, NBL infants, and GCT in England). Figure 2 illustrates 5‐year survival for most types of childhood cancers in Japan and England in 1993‐1996 and in 2005‐2008. Difference in 5‐year survival between countries narrowed from 1993‐1996 to 2005‐2008 for CML, lymphomas, CNS tumors, retinoblastoma, soft tissue sarcomas and RMS. In contrast, 5‐year survival was still less than 80% in both countries even in the most recent period for AML (Japan: 78%, England: 66%), CNS tumors (Japan: 59%, England: 57%), NBL children aged 1‐14 years (Japan: 75%, England: 57%), malignant bone tumors (Japan: 67%, England: 65%), soft tissue sarcomas (Japan: 68%, England: 73%) and RMS (Japan: 59%, England: 70%). Figure 3 shows the changes in 5‐year survival for each cancer type (except for other carcinomas and unspecified cancers) in each country from 1993‐1996 to 2005‐2008. Five‐year survival improved for most cancers except for renal tumors in Japan, and CNS tumors and NBL infants in England. Survival for CML dramatically improved in both countries (Japan: 67% to 100%, England: 44% to 84%).

**Table 4:** Overall 1‐y, 5‐y and 10‐y survival of childhood cancer (age 0‐14 y) by period of diagnosis and cancer type in Japan and England

	Japan							England						
	N at risk	1‐y	[95% CI]	5‐y	[95% CI]	10‐y	[95% CI]	N at risk	1‐y	[95% CI]	5‐y	[95% CI]	10‐y	[95% CI]
I. Leukemias														
1993‐1996	442	89.3	[86‐91.8]	71.2	[66.7‐75.2]	67.8	[63.2‐72]	1564	91.0	[89.5‐92.4]	76.0	[73.8‐78.1]	70.4	[68‐72.6]
1997‐2000	355	94.6	[91.7‐96.5]	77.7	[73‐81.7]	74.5	[69.6‐78.7]	1627	91.3	[89.8‐92.5]	78.5	[76.4‐80.4]	76.0	[73.9‐78]
2001‐2004	371	93.7	[90.7‐95.8]	80.1	[75.7‐83.9]	*73.8*	*[68.8*‐*78.2]*	1630	92.8	[91.4‐93.9]	84.2	[82.3‐85.9]	82.3	[80.4‐84.1]
2005‐2008	369	94.7	[91.8‐96.6]	82.9	[78.5‐86.4]	*79.8*	*[75.2*‐*83.6]*	1518	94.3	[93‐95.4]	88.1	[86.3‐89.6]	*84.6*	*[82.7*‐*86.3]*
ALL children aged 1‐14 y														
1993‐1996	269	92.5	[88.6‐95.1]	79.2	[73.8‐83.6]	74.1	[68.4‐79]	1195	95.7	[94.4‐96.7]	82.8	[80.6‐84.9]	76.8	[74.3‐79.1]
1997‐2000	224	96.8	[93.5‐98.5]	82.8	[77.2‐87.2]	79.1	[73.1‐83.9]	1226	94.6	[93.2‐95.7]	84.1	[81.9‐86]	81.3	[79‐83.4]
2001‐2004	236	97.0	[93.8‐98.5]	85.7	[80.4‐89.6]	*77.1*	*[70.8*‐*82.1]*	1222	95.3	[94‐96.4]	88.9	[87‐90.6]	86.9	[84.8‐88.6]
2005‐2008	225	97.3	[94‐98.8]	86.8	[81.6‐90.6]	*84.1*	*[78.5*‐*88.3]*	1155	97.6	[96.5‐98.3]	93.2	[91.5‐94.5]	*88.8*	*[86.8*‐*90.5]*
AML														
1993‐1996	104	86.4	[78.1‐91.7]	62.1	[52‐70.7]	61.1	[51‐69.7]	258	75.6	[69.9‐80.4]	55.0	[48.8‐60.9]	51.5	[45.3‐57.4]
1997‐2000	94	91.4	[83.6‐95.6]	68.6	[58‐77]	67.5	[56.9‐76]	256	82.0	[76.8‐86.2]	63.3	[57.1‐68.9]	61.7	[55.5‐67.4]
2001‐2004	95	88.1	[79.6‐93.3]	66.4	[55.7‐75]	*64.9*	*[53.5*‐*74.2]*	270	84.4	[79.5‐88.3]	66.6	[60.7‐71.9]	65.2	[59.1‐70.5]
2005‐2008	101	93.7	[86.6‐97.1]	77.9	[68.1‐85]	*72.6*	*[62.1*‐*80.7]*	216	79.2	[73.1‐84]	66.2	[59.5‐72.1]	*68.1*	*[61.8*‐*73.6]*
CML														
1993‐1996	21	90.5	[67‐97.5]	66.7	[42.5‐82.5]	66.7	[42.5‐82.5]	23	73.9	[50.9‐87.3]	43.5	[23.3‐62.1]	34.8	[16.6‐53.7]
1997‐2000	11	90.9	[50.8‐98.7]	72.7	[37.1‐90.3]	54.5	[22.9‐78]	42	73.8	[57.7‐84.6]	52.4	[36.4‐66.1]	52.4	[36.4‐66.1]
2001‐2004	11	90.9	[50.8‐98.7]	81.8	[44.7‐95.1]	*66.3*	*[32.9*‐*85.9]*	32	90.6	[73.7‐96.9]	84.4	[66.5‐93.2]	81.3	[62.9‐91.1]
2005‐2008	8	100		100		*87.5*	*[38.7*‐*98.1]*	25	100		84.0	[62.8‐93.7]	*88.7*	*[69*‐*96.2]*
II. Lymphomas														
1993‐1996	130	80.5	[72.6‐86.4]	72.7	[64.1‐79.6]	71.9	[63.3‐78.9]	480	90.4	[87.4‐92.7]	82.3	[78.6‐85.4]	80.6	[76.8‐83.9]
1997‐2000	104	96.1	[89.9‐98.5]	89.1	[81.2‐93.8]	87.2	[78.9‐92.3]	562	91.8	[89.2‐93.8]	87.2	[84.1‐89.7]	85.8	[82.6‐88.4]
2001‐2004	105	94.2	[87.6‐97.4]	90.4	[82.9‐94.7]	*87.4*	*[79.2*‐*92.5]*	528	92.2	[89.6‐94.2]	88.1	[85‐90.6]	86.5	[83.3‐89.2]
2005‐2008	102	92.9	[85.7‐96.5]	86.8	[78.3‐92.1]	*87.9*	*[79.7*‐*93]*	571	95.8	[93.8‐97.2]	91.2	[88.6‐93.3]	*90.1*	*[87.3*‐*92.4]*
III. CNS tumors														
1993‐1996	168	77.9	[70.8‐83.4]	50.9	[43.1‐58.2]	44.8	[37.2‐52.2]	818	78.5	[75.5‐81.1]	59.6	[56.2‐62.9]	55.8	[52.3‐59.1]
1997‐2000	155	80.6	[73.5‐86]	60.6	[52.5‐67.8]	53.2	[45‐60.7]	783	74.1	[70.9‐77]	57.8	[54.3‐61.2]	52.7	[49.1‐56.1]
2001‐2004	159	80.3	[73.2‐85.7]	56.7	[48.6‐64]	*51.8*	*[43*‐*59.9]*	794	77.8	[74.8‐80.6]	58.1	[54.5‐61.4]	53.5	[50‐56.9]
2005‐2008	163	84.3	[77.6‐89.1]	58.9	[50.8‐66.1]	*51.8*	*[43.8*‐*59.2]*	769	75.3	[72.1‐78.2]	57.2	[53.6‐60.6]	*52.3*	*[48.7*‐*55.7]*
IV. Neuroblastoma														
1993‐1996	203	96.0	[92.2‐98]	85.1	[79.4‐89.4]	84.1	[78.3‐88.5]	299	84.3	[79.6‐87.9]	57.8	[52‐63.2]	56.8	[51‐62.2]
1997‐2000	126	92.9	[86.7‐96.2]	83.3	[75.6‐88.8]	79.1	[70.9‐85.3]	311	85.9	[81.5‐89.3]	62.0	[56.3‐67.1]	58.7	[53‐64]
2001‐2004	147	96.6	[91.9‐98.6]	87.6	[81‐92]	*84.2*	*[77*‐*89.3]*	313	87.9	[83.7‐91]	64.9	[59.3‐69.9]	61.7	[56‐66.8]
2005‐2008	71	97.1	[88.7‐99.3]	79.3	[67.6‐87.2]	*85.4*	*[77*‐*90.8]*	278	90.3	[86.2‐93.2]	65.0	[59.1‐70.3]	*63.2*	*[57.4*‐*68.4]*
NBL infants (age <1 y)														
1993‐1996	140	97.9	[93.5‐99.3]	97.1	[92.6‐98.9]	97.1	[92.6‐98.9]	83	92.8	[84.6‐96.7]	86.7	[77.3‐92.4]	86.7	[77.3‐92.4]
1997‐2000	85	95.3	[87.9‐98.2]	92.9	[85‐96.8]	91.7	[83.3‐95.9]	84	89.3	[80.4‐94.3]	83.3	[73.4‐89.8]	83.3	[73.4‐89.8]
2001‐2004	75	100		98.6	[90.8‐99.8]	*96.6*	*[89.8*‐*98.9]*	116	94.0	[87.8‐97.1]	90.5	[83.5‐94.6]	90.5	[83.5‐94.6]
2005‐2008	14	100		100		*98.9*	*[92.5*‐*99.8]*	88	88.6	[79.9‐93.7]	82.9	[73.3‐89.3]	*87.4*	*[78.9*‐*92.7]*
NBL children aged 1‐14 y														
1993‐1996	63	91.9	[81.7‐96.6]	58.1	[44.8‐69.2]	54.8	[41.7‐66.2]	216	81.0	[75.1‐85.6]	46.8	[40‐53.2]	45.4	[38.6‐51.9]
1997‐2000	41	87.8	[73.2‐94.7]	63.4	[46.8‐76.1]	53.4	[37‐67.2]	227	84.6	[79.2‐88.7]	54.2	[47.5‐60.4]	49.7	[43.1‐56]
2001‐2004	72	93.0	[83.9‐97]	76.1	[64.3‐84.4]	*62.1*	*[46.7*‐*74.3]*	197	84.3	[78.4‐88.7]	49.7	[42.6‐56.5]	44.7	[37.6‐51.5]
2005‐2008	57	96.4	[86.2‐99.1]	74.5	[60.8‐84.1]	*73.4*	*[57.9*‐*84]*	190	91.1	[86‐94.3]	56.7	[49.3‐63.4]	*50.3*	*[43.1*‐*57.1]*
V. Retinoblastoma														
1993‐1996	38	100		89.5	[74.3‐95.9]	89.5	[74.3‐95.9]	149	99.3	[95.3‐99.9]	96.6	[92.1‐98.6]	96.6	[92.1‐98.6]
1997‐2000	31	96.8	[79.2‐99.5]	96.8	[79.2‐99.5]	93.5	[76.6‐98.3]	129	96.9	[91.9‐98.8]	94.5	[88.9‐97.4]	94.5	[88.9‐97.4]
2001‐2004	41	100		95.1	[81.9‐98.8]	*91.6*	*[76.1*‐*97.2]*	133	99.2	[94.8‐99.9]	98.5	[94.1‐99.6]	98.5	[94.1‐99.6]
2005‐2008	37	100		100		*100*		137	100		100		*99.2*	*[94.7*‐*99.9]*
VI. Renal tumors														
1993‐1996	33	93.9	[77.9‐98.4]	84.7	[67.1‐93.4]	81.6	[63.5‐91.3]	282	90.4	[86.3‐93.3]	81.2	[76.1‐85.3]	80.5	[75.4‐84.7]
1997‐2000	29	93.1	[75.1‐98.2]	86.2	[67.3‐94.6]	86.2	[67.3‐94.6]	274	94.9	[91.5‐96.9]	89.8	[85.5‐92.8]	88.3	[83.9‐91.6]
2001‐2004	32	100		87.0	[68.9‐94.9]	*86.3*	*[67.4*‐*94.6]*	307	93.1	[89.7‐95.5]	85.6	[81.2‐89.1]	85.6	[81.2‐89.1]
2005‐2008	24	100		82.6	[60.1‐93.1]	*86.5*	*[68*‐*94.7]*	299	92.6	[89‐95.1]	85.2	[80.6‐88.8]	*84.1*	*[79.5*‐*87.7]*
VII. Hepatic tumors														
1993‐1996	15	80.0	[50‐93.1]	73.3	[43.6‐89.1]	58.3	[29.3‐78.9]	50	82.0	[68.3‐90.2]	70.0	[55.3‐80.7]	70.0	[55.3‐80.7]
1997‐2000	28	73.8	[52.6‐86.6]	58.2	[37.3‐74.3]	58.2	[37.3‐74.3]	48	79.2	[64.7‐88.2]	64.6	[49.4‐76.3]	60.4	[45.2‐72.6]
2001‐2004	25	96.0	[74.8‐99.4]	92.0	[71.6‐97.9]	*73.8*	*[44.8*‐*89.1]*	65	83.1	[71.5‐90.2]	69.2	[56.5‐78.9]	64.6	[51.7‐74.9]
2005‐2008	25	76.0	[54.2‐88.4]	76.0	[54.2‐88.4]	*75.9*	*[54*‐*88.4]*	55	87.3	[75.1‐93.7]	81.8	[68.8‐89.8]	*75.3*	*[61.3*‐*84.9]*
VIII. Malignant bone tumors														
1993‐1996	47	87.2	[73.8‐94.1]	61.7	[46.3‐73.9]	57.0	[41.5‐69.7]	212	92.5	[88‐95.3]	61.8	[54.9‐68]	55.7	[48.7‐62]
1997‐2000	58	94.6	[84.3‐98.2]	67.9	[53.9‐78.4]	62.1	[48‐73.5]	206	88.3	[83.1‐92]	66.0	[59.1‐72]	59.2	[52.2‐65.6]
2001‐2004	51	94.0	[82.5‐98]	72.0	[57.4‐82.4]	*65.0*	*[50.1*‐*76.4]*	231	93.9	[90‐96.4]	58.9	[52.2‐64.9]	55.0	[48.3‐61.1]
2005‐2008	43	93.0	[79.9‐97.7]	67.4	[51.3‐79.3]	*63.3*	*[47.9*‐*75.3]*	225	92.4	[88.1‐95.2]	64.9	[58.3‐70.7]	*58.2*	*[51.2*‐*64.6]*
IX. Soft tissue sarcomas														
1993‐1996	64	89.1	[78.4‐94.6]	57.8	[44.8‐68.8]	53.0	[40.1‐64.4]	320	90.6	[86.9‐93.3]	69.0	[63.6‐73.7]	66.4	[60.9‐71.3]
1997‐2000	63	88.7	[77.8‐94.5]	61.3	[48‐72.1]	59.6	[46.4‐70.6]	294	87.1	[82.7‐90.4]	66.7	[61‐71.7]	62.9	[57.1‐68.2]
2001‐2004	61	95.0	[85.3‐98.4]	70.0	[56.7‐79.9]	*65.6*	*[52.5*‐*76]*	319	85.6	[81.2‐89]	63.1	[57.6‐68.2]	60.6	[55‐65.7]
2005‐2008	58	85.7	[73.5‐92.6]	67.9	[53.9‐78.4]	*56.3*	*[43*‐*67.7]*	297	87.2	[82.8‐90.5]	72.9	[67.4‐77.6]	*63.2*	*[57.5*‐*68.4]*
Rhabdomyosarcoma														
1993‐1996	36	88.9	[73.1‐95.7]	41.7	[25.6‐57]	38.7	[23.1‐54.1]	199	91.5	[86.6‐94.6]	67.6	[60.6‐73.7]	67.1	[60.1‐73.2]
1997‐2000	36	86.1	[69.8‐94]	47.2	[30.5‐62.3]	44.4	[28‐59.6]	164	86.6	[80.3‐91]	65.9	[58.1‐72.6]	62.8	[54.9‐69.7]
2001‐2004	35	94.1	[78.5‐98.5]	64.7	[46.3‐78.2]	*57.2*	*[37.8‐72.5]*	164	86.6	[80.3‐91]	56.5	[48.6‐63.7]	54.7	[46.7‐61.9]
2005‐2008	22	86.4	[63.4‐95.4]	59.1	[36.1‐76.2]	*48.5*	*[29.9‐64.8]*	157	88.5	[82.3‐92.6]	70.3	[62.4‐76.8]	*56.8*	*[48.7‐64]*
X. Germ cell tumors														
1993‐1996	80	92.5	[84‐96.6]	84.9	[74.9‐91.1]	84.9	[74.9‐91.1]	151	94.0	[88.9‐96.9]	86.8	[80.2‐91.2]	85.4	[78.7‐90.2]
1997‐2000	80	95.0	[87.1‐98.1]	91.2	[82.3‐95.7]	89.9	[80.8‐94.8]	142	96.5	[91.7‐98.5]	89.4	[83‐93.5]	89.4	[83‐93.5]
2001‐2004	73	98.6	[90.2‐99.8]	95.7	[87.1‐98.6]	*95.2*	*[85.7‐98.4]*	151	98.0	[94‐99.4]	93.4	[88‐96.4]	92.7	[87.2‐95.9]
2005‐2008	67	96.9	[88.1‐99.2]	95.3	[86‐98.4]	*92.1*	*[82‐96.7]*	173	96.0	[91.7‐98.1]	93.6	[88.8‐96.4]	*93.8*	*[88.8‐96.6]*
XI. Other carcinomas														
1993‐1996	29	93.1	[75.1‐98.2]	89.7	[71.3‐96.5]	86.1	[67‐94.5]	127	94.5	[88.8‐97.3]	85.8	[78.5‐90.8]	83.5	[75.8‐88.9]
1997‐2000	27	96.3	[76.5‐99.5]	92.6	[73.5‐98.1]	84.8	[64.5‐94]	126	92.9	[86.7‐96.2]	83.3	[75.6‐88.8]	81.7	[73.8‐87.5]
2001‐2004	31	96.6	[77.9‐99.5]	86.2	[67.3‐94.6]	*82.8*	*[63.4‐92.5]*	147	94.5	[89.4‐97.2]	87.7	[81.2‐92.1]	87.0	[80.4‐91.5]
2005‐2008	23	85.0	[60.4‐94.9]	80.0	[55.1‐92]	*75.6*	*[53.5‐88.2]*	151	92.7	[87.2‐95.9]	89.4	[83.2‐93.3]	*88.0*	*[81.7‐92.3]*
XII. Unspecified cancers														
1993‐1996	7	100		83.3	[27.3‐97.5]	83.3	[27.3‐97.5]	50	90.0	[77.6‐95.7]	85.9	[72.7‐93]	85.9	[72.7‐93]
1997‐2000	13	92.3	[56.6‐98.9]	76.9	[44.2‐91.9]	69.2	[37.3‐87.2]	33	87.9	[70.9‐95.3]	78.8	[60.6‐89.3]	75.8	[57.3‐87.1]
2001‐2004	12	91.7	[53.9‐98.8]	83.3	[48.2‐95.6]	*79.3*	*[48.5‐92.9]*	25	84.0	[62.8‐93.7]	72.0	[50.1‐85.5]	72.0	[50.1‐85.5]
2005‐2008	5	80.0	[20.4‐96.9]	60.0	[12.6‐88.2]	*66.3*	*[26.6‐88]*	50	100		89.8	[77.2‐95.6]	*89.2*	*[69.4‐96.5]*

Cohort approach (1‐y and 5‐y overall survival [OS] in 1993‐2008 in both countries, 10‐y OS in 1993‐2000 in Japan and in 1993‐2004 in England) and *period approach (10‐y OS in 2001‐2008 in Japan and in 2005‐2008 in England)*. ALL, acute lymphoblastic leukemias; AML, acute myeloid leukemias; CML, chronic myeloid leukemia; CNS, central nervous system; NBL, neuroblastoma.

## 4 DISCUSSION

In this study, we compared incidence and trends in survival for each childhood cancer type in Japan and England. Incidence of all childhood cancers combined decreased in Japan throughout 1993‐2010 (Table 2, Figure S1), whereas in England incidence for all cancers combined was stable from 1999‐2004 to 2005‐2010, after a slight increase in the earlier period. However, analysis of the incidence by cancer type showed that the trends in cancer‐specific incidence hardly varied in each county, except for neuroblastoma in Japan (Table 2). The apparent drop in incidence for neuroblastoma in Japan was probably due to the cessation of the national screening program for neuroblastoma, which had been conducted as urine tests for all infants at 6 months of age since 1985.^20^, ^21^ The Ministry of Health, Labour and Welfare terminated the program in 2004 on the basis of the self‐healing potential of infant NBL and the negative effects of screening on mortality.^22^ In the most recent period, after the cessation of this screening program in Japan, incidence for all cancers was higher in England than in Japan (ASR 2005‐2010 139 vs 116). The incidence of many cancer types differed between Japan and England. In England, incidence of HL, renal tumors and Ewing sarcomas was more than double that of Japan. Previous studies have shown racial differences in incidence for these cancers.^23^, ^24^, ^25^, ^26^, ^27^, ^28^, ^29^ Etiological factors of HL have been suggested by the bimodal age distribution, by elevated risks in males, by the occurrence of Epstein–Barr virus in HL tumor cells, and by identifying inherited susceptibility genes; however, the mechanism by which racial differences in incidence for HL occur is still unclear. Regarding renal tumors, previous studies which reported on differences in age distribution between countries for Wilms tumor showed the peak age for occurrence in East Asia to be infants (age <1 year), but among Caucasians in the USA the peak occurrence was older.^24^, ^25^ Our study supports these findings (Table S2 and Figure S2). Some other reports show differences in epigenetic factors in Wilms tumor between Japanese and Caucasians.^26^, ^27^ For Ewing sarcomas, one report showed that Japanese Ewing sarcoma patients have a higher frequency of loss of chromosome 19 than European Caucasian patients.^28^ However, these tumors are rare and their etiology has not been sufficiently investigated to explain these differences in incidence.^29^ Regarding the higher incidence of AML in Japan, Bessho reported the mis‐classification of ALL to ANLL (AML), which overestimated the proportion of ANLL in the 1970s.^30^ However, nowadays, diagnosis of leukemia has become much more accurate and the proportion of unknown leukemia subtype was only approximately 5% in our data (Table 3). The ALL:AML ratio in our data was 2.4:1, which is similar to that found in the report of the Japanese pediatric leukemia study group (JPLSG), containing information on molecular abnormalities collected by pediatric oncologists (ALL:AML = 2.8:1).^31^ On the IICC‐3 website, the ASR of AML was around 10 per million person‐years in Japan and Korea,^32^ whereas the figure was around 7 per million person‐years in an Austria‐based study.^19^ The CONCORD‐2 study on cancer survival reported higher proportions of AML in Asia than in Europe.^7^ In the US data, there are no large racial differences in incidence for AML.^33^ Further research will be needed to clarify whether the differences we have observed are due to underlying ethnic difference in the incidence of AML.

When comparing incidence for each subgroup, the proportion of “unspecified” histology within each cancer group should be taken into account (Table 3). The proportion of “unspecified” lymphomas (ICCC‐3 II‐e; 18%) or “unspecified” CNS tumors (ICCC‐3 III‐f; 16%) in Japan was over 10% within each cancer group in the total period (1993‐2010), although it decreased to under 10% in the most recent periods (data not shown).

Five‐year survival for most cancer types improved in both Japan and England. (Table 4). For example, survival of childhood leukemias improved constantly in both countries throughout 1993‐2008. Risk stratification and improvement in clinical trials/treatment may have contributed to the improvement in survival for ALL (children aged 1‐14 years) and AML in both countries.^31^, ^34^, ^35^, ^36^ Survival in CML improved dramatically in both countries after the introduction of the tyrosine kinase inhibitor (TKI) imatinib (trade name Gleevec) approved by the US FDA, Japan and the UK in 2001 (Figure 3).^37^ This is an impressive example of an effective therapy changing the survival of patients dramatically. For survivors, however, careful, long‐term follow‐up is needed because several case reports have described growth impairment of pediatric CML patients as an adverse effect of imatinib.^38^, ^39^ Differences in 5‐year survival in some cancer types (CML, lymphomas, CNS tumors, retinoblastoma, soft tissue sarcomas and rhabdomyosarcoma) between countries seem to be narrowing (Figure 2). This may be the result of recent international collaboration between countries. However, even for the most recent periods, 5‐year survival for several cancer types (AML, CNS tumors, NBL [children aged 1‐14 years], soft tissue sarcomas, malignant bone tumors) remains <80% in both countries. To improve survival for patients with these cancer types, we should target research at developing new drugs and improving treatment protocols. Five‐year survival of renal tumors in Japan, NBL under 1 year of age and CNS tumors in England decreased in the more recent period (Figure 3). One possible reason for the latter is that more cases of pilocytic astrocytoma could have been coded and classified as astrocytoma NOS or glioma NOS (both with the malignant behavior code) in the earlier years.

### 4.1 Cancer strategy for childhood cancer

Since 1974, the Japanese Government has subsidized medical expenses for children and adolescents under 18 years of age with cancer.^40^ The *National Cancer Control Act* in Japan was established in 2006, initially focusing on major adult cancers. The second cancer control plan in 2012 first raised the issue of care for children and young cancer patients. Fifteen hospitals were designated as childhood cancer care hospitals in 2012 to increase centralization and cooperation between all the hospitals in Japan.

In the UK, the first National Health Service cancer control plan started in 2000. “Improving outcomes in children and young people with cancer” was published as national guidance for cancer services by the National Institute for Health and Clinical Excellence in 2005. There are 20 specialized hospitals for childhood cancer, known as principal treatment centers, and over 80 shared care centers, known as Paediatric Oncology Shared Care Units. In Europe, similar but less detailed standards of care for children with cancer were published in 2013,^41^ with an international survey of the extent of their implementation published in 2016.^42^ All standards recommend coordinated patient care and international collaboration in research.

In this study, we looked at the trends in cancer incidence and survival in children over a 15‐year period for Japan and England, by using population‐based cancer registry data and compared them during the same periods for each cancer type. One limitation of our study is the small number of records available in the Japanese dataset. Prefectural cancer registry data were only available for 6 prefectures, representing 14% of the total population,^10^ because other registries did not have such long‐term data with patients’ vital status information. In 2013, a law for cancer registration was established in Japan and a nationwide cancer registration system started in 2016. Another limitation was the divergence between 5‐year survival and 10‐year survival (higher survival in 10‐year survival than 5‐year survival) in several cancers because we used the period approach to predict 10‐year survival in recent periods. To improve surveillance and comparability, we need to keep collecting data widely and precisely, and follow up patients’ vital status in the long term.

In conclusion, the incidence rates of the majority of childhood cancers differed significantly between Japan and England. Some of these differences are explained by differences in national screening practices (infant neuroblastoma) and known differences in the genetics of Wilms tumor. Further research is needed to explore how much these variations in incidence are due to genetic susceptibility and/or environmental etiological factors. Regarding survival, an improvement was observed for most cancer types during the period 1993‐2008 in both countries. The increase was particularly notable for CML, following the introduction of effective, targeted treatment. Variations in survival may be due to differences in the tumor biology or in the treatment or in the health‐care service quality. The role for these factors will be further investigated through planned collaborative clinical and translational research between the 2 countries. Survival remained poor for 5 main cancer types, even in recent periods. This emphasizes the continuing need for new drug development while other opportunities for survival improvement should not be ignored, such as a better understanding of the potential impact of health‐care service organization and quality on survival, or through clinical studies to optimize the use of current treatments.

## CONFLICT OF INTEREST

The authors have no conflicts of interest to declare.
