# Supplementary material for: Childhood cancer incidence and survival in Japan and England: A population‐based study (1993‐2010)
Source: Cancer Sci. 2017 Dec 26;109(2):422–34. doi: 10.1111/cas.13457 (PMC5797810; doi:10.1111/cas.13457)
Supplement: Supplementary file 1 [file CAS-109-422-s001.docx]

**Table S1**. **ICD-O3 code for each subgroup**

| Subgroup |  | ICD-O3 code | |
| --- | --- | --- | --- |
|  |  | Morphology | Topography |
| Acute lymphoblastic leukaemias (ALL) |  | 9835-9837 | C00.0-C80.9 |
| Acute myeloid leukaemias (AML) |  | 9840, 9861, 9866, 9867, 9870-9874, 9891, 9895-9897, 9910, 9920, 9931 | C00.0-C80.9 |
| Chronic myeloid leukaemia (CML) |  | 9863, 9875,9876 | C00.0-C80.9 |
| Hodgkin lymphomas |  | 9650–9655, 9659, 9661–9665, 9667 | C00.0-C80.9 |
| non-Hodgkin lymphomas |  | 9591, 9670, 9671, 9673, 9675, 9678–9680, 9684, 9687, 9689–9691, 9695, 9698–9702, 9705, 9708, 9709, 9714, 9716–9719, 9727–9729, 9731–9734, 9760–9762, 9764–9769, 9970 | C00.0-C80.9 |
| Astrocytoma |  | 9380 | C72.3 |
|  |  | 9384, 9400–9411, 9420, 9421–9424, 9440-9442 | C00.0-C80.9 |
| Medulloblastoma |  | 9470-9474 | C00.0-C80.9 |
| Wilms Tumour |  | 8960 | C00.0-C80.9 |
| Hepatoblastoma |  | 8970 | C00.0-C80.9 |
| Osteosarcaomas |  | 9180–9187, 9191–9195, 9200 | C40.0–C41.9, C76.0–C76.8,C80.9 |
| Ewing sarcomas (both bone and soft tissue) |  | 9260, 9365 | C00.0-C80.9 |
|  |  | 9364 | C00.0-C69.9, C73.9-C80.9 |
| Rhabdomyosarcoma |  | 8900–8905, 8910, 8912, 8920, 8991 | C00.0-C80.9 |
| Germ cell tumours |  |  |  |
| Intracranial |  | 9060–9065, 9070–9072, 9080–9085, 9100, 9101 | C70.0–C72.9, C75.1–C75.3 |
| Extracranial |  | 9060–9065, 9070–9072, 9080–9085, 9100–9105 | C00.0–C55.9, C57.0–C61.9, C63.0-C69.9, C73.9–C75.0, C75.4–C76.8, C80.9 |
| Gonadal |  | 9060–9065, 9070–9073, 9080–9085, 9090, 9091, 9100, 9101 | C56.9, C62.0–C62.9 |

**Table S2**. **Age-, sex-, specific incidence rates (per million person-years) of Wilms tumor, hepatoblastoma, and germ cell tumours, Japan and England**

|  |  | **Male & Female** | | | | | | | | | | **Male** | | | | | | | | | | **Female** | | | | | | | | | |
| --- | --- | --- | --- | --- | --- | --- | --- | --- | --- | --- | --- | --- | --- | --- | --- | --- | --- | --- | --- | --- | --- | --- | --- | --- | --- | --- | --- | --- | --- | --- | --- |
|  |  | 0 | | 1-4 | | 5-9 | | 10-14 | | 0-14 | | 0 | | 1-4 | | 5-9 | | 10-14 | | 0-14 | | 0 | | 1-4 | | 5-9 | | 10-14 | | 0-14 | |
|  |  | *n* | rate | *n* | rate | *N* | rate | *n* | rate | *n* | rate | *n* | rate | *n* | rate | *n* | rate | *n* | rate | *n* | rate | *n* | rate | *n* | rate | *n* | rate | *n* | rate | *n* | rate |
| **Wilms tumor** | | |  |  |  |  |  |  |  |  |  |  |  |  |  |  |  |  |  |  |  |  |  |  |  |  |  |  |  |  |  |
| Japan |  | 22 | 7.9 | 71 | 6.2 | 14 | 0.9 | 6 | 0.4 | 113 | 2.5 | 11 | 7.7 | 37 | 6.4 | 7 | 0.9 | 2 | 0.2 | 57 | 2.5 | 11 | 8.1 | 34 | 6.1 | 7 | 0.96 | 4 | 0.51 | 56 | 2.5 |
| England |  | 130 | 11.8 | 783 | 17.8 | 235 | 4.2 | 42 | 0.7 | 1190 | 7.1 | 67 | 11.9 | 372 | 16.5 | 102 | 3.6 | 22 | 0.8 | 563 | 6.6 | 63 | 11.7 | 411 | 19.2 | 133 | 4.9 | 20 | 0.7 | 627 | 7.7 |
| **Hepatoblastoma** | | | |  |  |  |  |  |  |  |  |  |  |  |  |  |  |  |  |  |  |  |  |  |  |  |  |  |  |  |  |
| Japan |  | 37 | 13.3 | 48 | 4.2 | 12 | 0.8 | 3 | 0.2 | 100 | 2.2 | 20 | 14.0 | 29 | 5.0 | 9 | 1.2 | 2 | 0.2 | 60 | 2.6 | 17 | 12.5 | 19 | 3.4 | 3 | 0.41 | 1 | 0.13 | 40 | 1.8 |
| England |  | 77 | 7.0 | 113 | 2.6 | 12 | 0.2 | 9 | 0.2 | 211 | 1.3 | 39 | 6.9 | 71 | 3.2 | 11 | 0.4 | 7 | 0.2 | 128 | 1.5 | 38 | 7.1 | 42 | 2.0 | 1 | 0.04 | 2 | 0.07 | 83 | 1.0 |
| **Intracranial GCT** | | | |  |  |  |  |  |  |  |  |  |  |  |  |  |  |  |  |  |  |  |  |  |  |  |  |  |  |  |  |
| Japan |  | 10 | 3.6 | 6 | 0.5 | 39 | 2.6 | 98 | 6.1 | 153 | 3.4 | 6 | 4.2 | 4 | 0.7 | 27 | 3.5 | 71 | 8.6 | 108 | 4.7 | 4 | 2.9 | 2 | 0.4 | 12 | 1.6 | 27 | 3.4 | 45 | 2.0 |
| England |  | 4 | 0.4 | 7 | 0.2 | 40 | 0.7 | 109 | 1.9 | 160 | 1.0 | 2 | 0.4 | 3 | 0.1 | 24 | 0.8 | 86 | 3.0 | 115 | 1.3 | 2 | 0.4 | 4 | 0.2 | 16 | 0.6 | 23 | 0.8 | 45 | 0.6 |
| **extracranial GCT** | | | |  |  |  |  |  |  |  |  |  |  |  |  |  |  |  |  |  |  |  |  |  |  |  |  |  |  |  |  |
| Japan |  | 22 | 7.9 | 17 | 1.5 | 4 | 0.3 | 12 | 0.7 | 55 | 1.2 | 5 | 3.5 | 6 | 1.0 | 1 | 0.1 | 9 | 1.1 | 21 | 0.9 | 17 | 12.5 | 11 | 2.0 | 3 | 0.4 | 3 | 0.4 | 34 | 1.5 |
| England |  | 78 | 7.1 | 70 | 1.6 | 12 | 0.2 | 16 | 0.3 | 176 | 1.1 | 27 | 4.8 | 20 | 0.9 | 3 | 0.1 | 12 | 0.4 | 62 | 0.7 | 51 | 9.5 | 50 | 2.3 | 9 | 0.3 | 4 | 0.1 | 114 | 1.4 |
| **gonadal GCT** | | |  |  |  |  |  |  |  |  |  |  |  |  |  |  |  |  |  |  |  |  |  |  |  |  |  |  |  |  |  |
| Japan |  | 24 | 8.6 | 31 | 2.7 | 22 | 1.5 | 55 | 3.4 | 132 | 2.9 | 21 | 14.7 | 28 | 4.8 | 2 | 0.3 | 1 | 0.1 | 52 | 2.2 | 2 | 1.5 | 4 | 0.7 | 20 | 2.7 | 54 | 6.9 | 80 | 3.6 |
| England |  | 41 | 3.7 | 79 | 1.8 | 48 | 0.9 | 170 | 3.0 | 338 | 2.0 | 34 | 6.0 | 62 | 2.8 | 3 | 0.1 | 32 | 1.1 | 131 | 1.5 | 7 | 1.3 | 17 | 0.8 | 45 | 1.7 | 138 | 5.0 | 207 | 2.5 |

GCT, germ cell tumours

**Fig. S1**

1. **Trends in age-standardised incidence rate of all childhood cancers in Japan and England**

**b. Trends in incidence for neuroblastoma (age 0, 1-14, 0-14 years) in Japan and England**

**
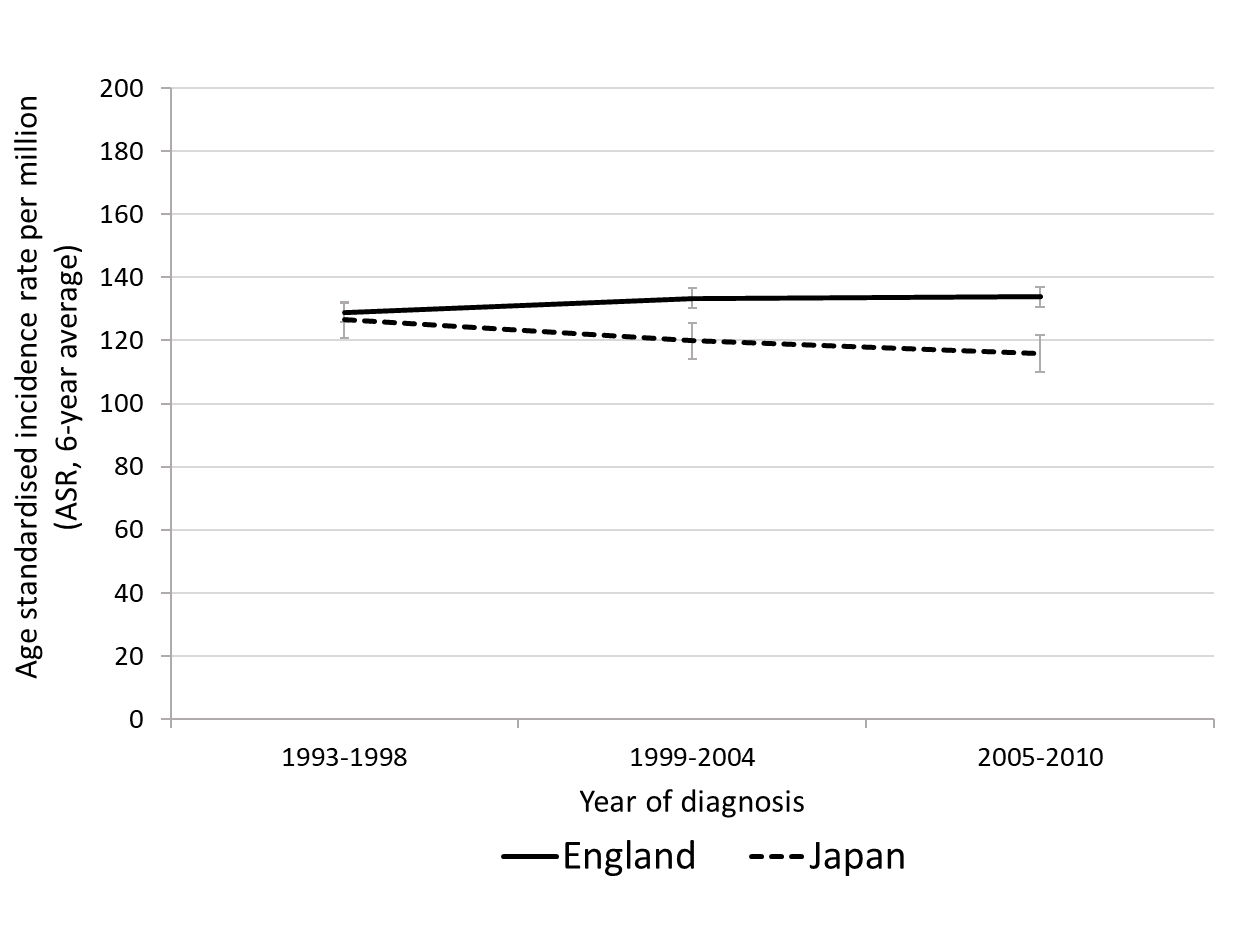

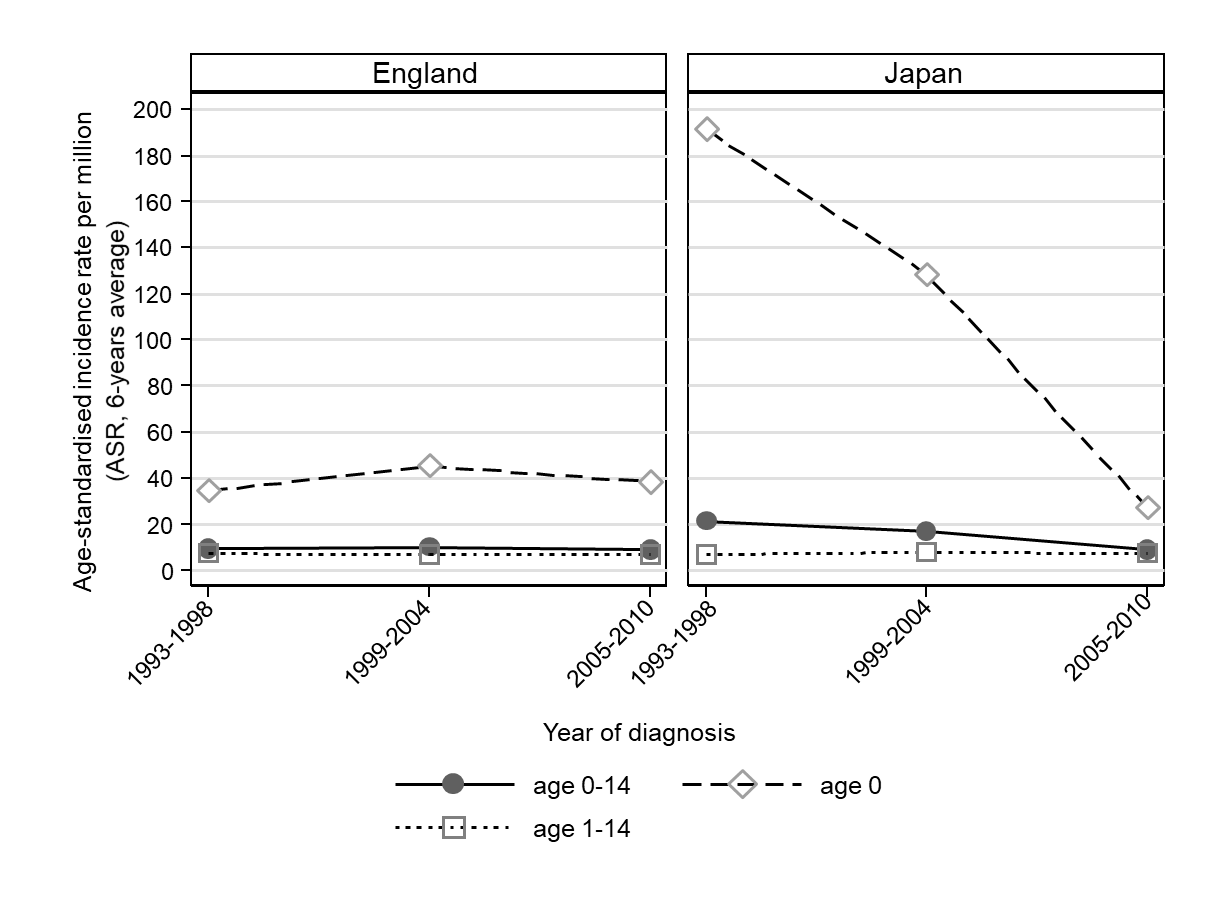
**

**Fig. S2. Age-distribution of incidence of Wilms tumor, hepatoblastoma, and germ cell tumours between Japan and England (both sexes)**
